# Successful Limb Salvage in MRSA Bacteremic Septic Charcot Midfoot Using Continuous Local Antibiotic Perfusion and Circular External Fixation: A Case Report

**DOI:** 10.3390/clinpract16060108

**Published:** 2026-06-09

**Authors:** Koji Nozaka, Naohisa Miyakoshi

**Affiliations:** Department of Orthopaedic Surgery, Akita University Graduate School of Medicine, 1-1-1 Hondo, Akita 010-8543, Japan

**Keywords:** Charcot foot, MRSA, external fixation, limb salvage, antibiotic perfusion

## Abstract

**Background**: Septic Charcot neuroarthropathy is a limb- and life-threatening condition characterized by the coexistence of neuropathic joint destruction and infection. In patients with severe systemic compromise, major amputation is often considered inevitable. **Case Presentation**: A 47-year-old man with untreated diabetes mellitus presented with progressive painless swelling of the left foot. He had morbid obesity (120 kg, 165 cm; body mass index 44.1 kg/m^2^), severe hypoalbuminemia, and chronic kidney disease associated with nephrotic syndrome. Laboratory tests showed marked inflammation and poor glycemic control, and blood cultures were positive for methicillin-resistant Staphylococcus aureus (MRSA). Radiographs and computed tomography demonstrated destructive changes involving the talonavicular and subtalar joints, consistent with septic Charcot neuroarthropathy involving the midfoot. Because of sepsis, pulmonary edema, and heart failure, below-knee amputation was proposed at the referring hospital. However, limb salvage was attempted using aggressive debridement, continuous local antibiotic perfusion (CLAP; gentamicin 1200 μg/mL) administered for 14 days, and temporary circular external fixation. Serum gentamicin concentrations and renal function were regularly monitored to ensure systemic safety and avoid nephrotoxicity. **Results**: Repeat irrigation and final debridement were performed 20 days after the index surgery, at which time the external fixator was removed and intraoperative cultures were negative. The patient was discharged 2 months after surgery without evidence of recurrent infection. At 4-year follow-up, no recurrence had occurred, and the patient was able to walk independently. **Conclusions**: Limb salvage may be feasible even in severely compromised patients with septic Charcot midfoot and MRSA bacteremia when aggressive debridement, CLAP, and temporary external fixation are combined with careful systemic safety monitoring. This case suggests that limb salvage may be considered in selected high-risk patients, although further studies are required.

## 1. Background

Charcot neuroarthropathy is a progressive destructive disorder associated with peripheral neuropathy, most commonly in patients with diabetes mellitus [1,2,3]. Because pain is often minimal or absent despite substantial osseous and joint destruction, diagnosis is frequently delayed [4]. Delayed recognition worsens structural collapse and increases the risks of ulceration, infection, and amputation [5,6].

When infection coexists with Charcot neuroarthropathy, diagnosis and treatment become considerably more difficult. Elevated inflammatory markers alone are insufficient to distinguish superimposed infection from active Charcot arthropathy [7]. Current guidelines emphasize the importance of early diagnosis and prompt infection control in diabetes-related foot infections [8,9].

External fixation has been reported as an effective limb-salvage strategy in Charcot foot complicated by osteomyelitis because it provides temporary mechanical stability while preserving soft-tissue access and avoiding internal hardware in an infected field [10,11,12,13,14,15]. In addition, chronic kidney disease and other severe systemic comorbidities are associated with higher risks of amputation and mortality in diabetic foot disease [16,17,18,19].

Continuous local antibiotic perfusion (CLAP) has recently emerged as a promising adjunctive technique for difficult musculoskeletal infections by enabling high local antibiotic concentrations at the site of infection [20,21]. We report a case of septic Charcot midfoot involving the talonavicular and subtalar joints with MRSA bacteremia in a severely compromised patient, successfully managed with staged debridement, CLAP, and temporary circular external fixation.

## 2. Case Presentation

A 47-year-old man noticed progressive swelling of the left foot in mid-December without pain. In late January, he developed fever, inability to walk, and poor oral intake and was initially evaluated at an outside hospital.

His comorbidities included untreated diabetes mellitus, morbid obesity, severe hypoalbuminemia, and chronic kidney disease associated with nephrotic syndrome. On presentation, his body weight was 120 kg and his height was 165 cm, corresponding to a body mass index of 44.1 kg/m^2^. Laboratory findings showed a white blood cell count of 14,000/μL and a C-reactive protein level of 9.8 mg/dL. Glycemic control was markedly poor, with an HbA1c of 12.9% and blood glucose of 469 mg/dL. Serum albumin was 1.0 g/dL, serum creatinine was 2.1 mg/dL, and the estimated glomerular filtration rate was 31 mL/min/1.73 m^2^. Blood cultures obtained at the outside hospital were positive for MRSA.

**Figure 1:**
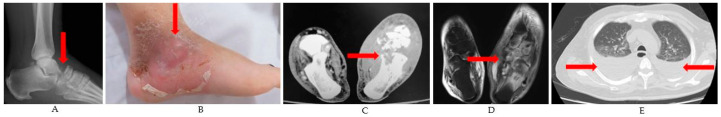
Initial clinical and radiographic findings at the outside hospital. (**A**) Lateral radiograph showing severe destruction of the talonavicular and subtalar joints (red arrow). (**B**) Clinical appearance showing medial ankle redness and swelling consistent with severe local inflammation (red arrow). (**C**) Computed tomography demonstrating destructive changes in the talonavicular and subtalar joints (red arrow). (**D**) T2-weighted magnetic resonance imaging demonstrating high-signal intensity in the soft tissue, consistent with abscess formation and severe inflammatory changes (red arrow). (**E**) Chest computed tomography demonstrating pulmonary edema associated with systemic deterioration (red arrow).

Plain radiographs demonstrated severe destructive changes involving the talonavicular and subtalar joints (Figure 1A). Despite the extent of osseous destruction, pain was not a prominent symptom, suggesting neuropathic arthropathy with superimposed infection. Clinical examination showed marked medial ankle redness and swelling consistent with severe local inflammation (Figure 1B). Computed tomography demonstrated destructive changes involving the talonavicular and subtalar joints (Figure 1C). T2-weighted magnetic resonance imaging demonstrated high-signal intensity in the soft tissue, consistent with abscess formation and severe inflammatory changes (Figure 1D). The patient developed sepsis accompanied by pulmonary edema and heart failure, and chest computed tomography demonstrated pulmonary edema associated with systemic deterioration (Figure 1E). Intravenous antibiotic therapy was initiated, but the clinical response was insufficient, and below-knee amputation was proposed. The patient was transferred to our institution for limb salvage.

These findings illustrate severe septic Charcot neuroarthropathy at initial presentation.

### 2.1. Surgical Management

**Figure 2:**
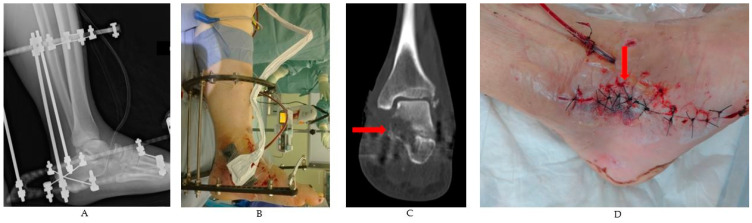
Early postoperative course during continuous local antibiotic perfusion and temporary external fixation. (**A**) Lateral radiograph obtained after the initial surgery demonstrating stabilization of the talonavicular and subtalar joints with external fixation. (**B**) Clinical appearance during CLAP therapy showing persistent but improving local inflammation of the medial ankle. (**C**) Computed tomography images demonstrating postoperative changes following curettage of infected bone in the talonavicular and subtalar joints (red arrow). (**D**) Clinical appearance at postoperative day 14 showing marked improvement of local inflammatory signs following debridement and CLAP therapy (red arrow).

Emergency surgery was performed through a medial approach to the ankle and foot. Extensive debridement of infected and necrotic tissue was undertaken. Sequestra and necrotic bone involving the medial talus, calcaneus, and navicular were removed. Intraoperative cultures obtained during the initial surgery were positive for methicillin-resistant *Staphylococcus aureus* (MRSA) from the talonavicular joint. Because of the severe local infection and the need for temporary mechanical stabilization, circular external fixation was applied, and postoperative radiographs demonstrated stabilization of the talonavicular and subtalar joints (Figure 2A).

The temporary circular external fixator consisted of a two-ring construct with one distal tibial ring and one foot ring. The foot ring was stabilized using two transosseous calcaneal wires and one midfoot transosseous wire, providing temporary stabilization across the ankle, subtalar, Chopart, and Lisfranc joints, including the infected talonavicular joint. Weight-bearing during fixation was permitted to reduce complications associated with prolonged immobilization, such as pneumonia, worsening heart failure, and deep vein thrombosis. Pin-site management was performed using an open-shower protocol while carefully avoiding dislodgement of the CLAP tubing and NPWT dressing.

After debridement, CLAP was initiated using gentamicin at 1200 μg/mL. In this case, CLAP was implemented using a combined system of continuous antibiotic perfusion and negative pressure wound therapy (NPWT). Antibiotics were delivered through intra-soft tissue perfusion (iSAP), while NPWT facilitated continuous drainage of exudate, hematoma, and residual antibiotics, thereby maintaining local antibiotic turnover. Gentamicin was prepared as 60 mg in 50 mL of normal saline, corresponding to a concentration of 1200 μg/mL, and was continuously infused at a rate of 2 mL/h. Continuous perfusion allowed sustained high local antibiotic concentrations while minimizing systemic exposure. The duration of CLAP therapy in this case was 14 days, which was determined based on clinical response, including resolution of local inflammatory findings and normalization of laboratory markers. Serum gentamicin concentrations and renal function parameters were regularly monitored to ensure systemic safety and avoid nephrotoxicity. Serum gentamicin concentrations measured on postoperative days 3, 7, and 14 remained below 1 μg/mL. In previous reports, serum gentamicin levels exceeding 1.0 μg/mL have been associated with an increased risk of nephrotoxicity, and therefore careful monitoring and dose adjustment are recommended.

Intravenous daptomycin was administered at 6 mg/kg once daily for 6 weeks as systemic anti-MRSA therapy. Daptomycin was selected instead of vancomycin because of the patient’s pre-existing renal dysfunction and concern regarding vancomycin-associated nephrotoxicity. During CLAP therapy, persistent but improving local inflammation of the medial ankle was observed (Figure 2B), and computed tomography demonstrated postoperative changes following curettage of infected bone in the talonavicular and subtalar joints (Figure 2C). Local redness, swelling, and heat gradually improved after surgery, with marked improvement evident by postoperative day 14 (Figure 2D). At that time, the white blood cell count and C-reactive protein level had normalized.

Twenty days after the index procedure, repeat irrigation and final debridement were performed. At that time, the external fixator was removed, and intraoperative cultures were negative for MRSA. Based on the resolution of local inflammatory signs, normalization of inflammatory markers, and negative intraoperative cultures at the second-stage surgery, infection control was considered to have been achieved. After fixator removal, immobilization was maintained using a splint for 2 weeks, followed by protected weight-bearing with a patellar tendon-bearing (PTB) orthosis.

### 2.2. Outcome and Follow-Up

The overall clinical course is summarized in Table 1.

**Figure 3:**
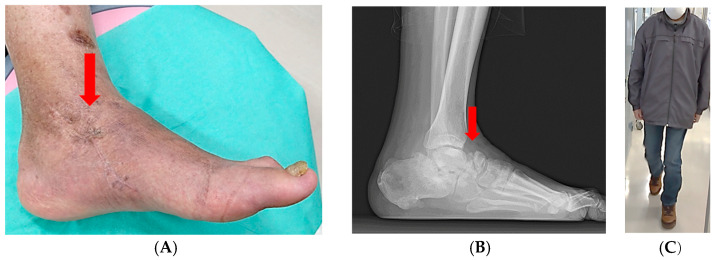
Long-term outcome at 4-year follow-up. (**A**) Clinical appearance showing the medial ankle without signs of active inflammation (red arrow). (**B**) Lateral radiograph demonstrating residual Charcot-type destructive changes involving the talonavicular and subtalar joints without evidence of recurrent infection (red arrow). (**C**) Clinical outcome demonstrating independent ambulation without reliance on orthotic support. Limb salvage was maintained with no recurrence of infection at 4 years, representing sustained infection-free survival in a severely compromised host.

The patient was discharged 2 months after the initial surgery without evidence of recurrent infection. At 4-year follow-up, no recurrence of infection had occurred. Clinical examination showed the medial ankle without signs of active inflammation (Figure 3A). The patient had achieved marked weight loss, from 120 kg to 65 kg. Radiographs showed residual Charcot-type destructive changes involving the talonavicular and subtalar joints but no evidence of uncontrolled recurrent infection (Figure 3B).

At final follow-up, the AOFAS score was 90 points, indicating a favorable functional outcome despite underlying neuropathy. Clinically, the patient was able to ambulate independently without pain using regular footwear, without a noticeable limp (Figure 3C). Although a patellar tendon-bearing (PTB) orthosis was prescribed, the patient did not use it during follow-up and was able to maintain independent ambulation in daily life.

These findings support meaningful functional limb salvage as an alternative to amputation.

## 3. Discussion

This case highlights several clinically important aspects in the management of septic Charcot neuroarthropathy involving the talonavicular and subtalar joints in a severely compromised host.

First, Charcot neuroarthropathy presents a diagnostic challenge. Patients may show severe structural destruction with minimal pain due to underlying neuropathy [1,2,3]. Because clinical features overlap with infection, misdiagnosis and delayed diagnosis are common and are associated with worse outcomes [4,5,6]. In this case, MRSA bacteremia, systemic deterioration, imaging findings, and intraoperative cultures supported the diagnosis of superimposed infection, although histopathological confirmation was not obtained. We considered the primary pathology in this case to be neuropathic Charcot collapse complicated by secondary deep infection, based on the painless presentation despite severe joint destruction, the distribution of collapse involving the talonavicular and subtalar joints, the patient’s history of recurrent foot infections associated with minor skin injuries, and the presence of MRSA bacteremia and positive intraoperative cultures.

Second, inflammatory markers alone are insufficient to distinguish infection from active Charcot arthropathy [7]. Therefore, diagnosis requires integration of clinical, imaging, microbiological, and intraoperative findings. In the present case, the combination of abscess formation on magnetic resonance imaging, MRSA bacteremia, positive intraoperative cultures from the talonavicular joint, and necrotic bone observed during debridement supported the diagnosis of septic Charcot neuroarthropathy with deep infection rather than uncomplicated Charcot collapse alone.

Third, this case highlights the complexity of treatment decision-making in medically complex patients with septic Charcot neuroarthropathy. In diabetic foot disease, chronic kidney disease and severe systemic comorbidities are associated with increased mortality and amputation risk [16,17,18,19,22,23]. In such patients, major amputation is often considered a reliable option. In the present case, however, severe malnutrition, renal dysfunction, cardiopulmonary instability, and extreme obesity increased perioperative risk, and a staged limb-salvage approach was selected. This case suggests that limb salvage may be considered in selected high-risk patients when infection control and temporary stabilization are achieved, although this observation remains hypothesis-generating.

Fourth, the treatment strategy relied on three principles: aggressive source control, temporary mechanical stabilization, and high-concentration local antibiotic delivery. External fixation provided stability while preserving soft-tissue access and avoiding internal implants in an infected field [10,11,12,13,14,15]. In this case, it was used as a temporary adjunct during infection control and was removed after microbiological and clinical resolution. This differs from conventional strategies that rely on prolonged fixation for definitive reconstruction. Temporary fixation may reduce pin-related complications and systemic burden, particularly in medically fragile patients, while maintaining sufficient stability during the critical infection-control period.

Fifth, CLAP was an important adjunctive component of the treatment strategy. Unlike conventional local antibiotic delivery methods such as antibiotic-loaded spacers or beads, which provide static release, CLAP enables continuous low-flow perfusion combined with drainage via NPWT. This allows sustained antibiotic turnover at the infection site. The rationale for CLAP is based on biofilm biology, where bacterial eradication requires antibiotic concentrations far exceeding conventional minimum inhibitory concentrations [24]. The use of high-concentration gentamicin was intended to achieve levels approaching the minimal biofilm eradication concentration. However, systemic absorption may occur, and elevated serum levels or acute kidney injury have been reported [25,26]. Therefore, CLAP requires careful pharmacokinetic monitoring. In this case, monitoring of serum gentamicin levels and renal function enabled safe application without nephrotoxicity despite chronic kidney disease.

Finally, the long-term outcome is clinically meaningful but should be interpreted cautiously. Infection-free survival for 4 years with independent ambulation is notable in a severely compromised patient. However, baseline preoperative functional status was unavailable, gait quality was assessed mainly qualitatively, and no patient-reported outcome measures were included. Therefore, the AOFAS score alone should not be overinterpreted as definitive evidence of functional recovery. The marked postoperative weight loss and improvement in systemic condition were considered multifactorial and likely reflected the combined effects of metabolic management, rehabilitation, recovery from systemic illness, and lifestyle modification after treatment. These systemic improvements may also have contributed to the favorable long-term outcome.

Overall, this case emphasizes clinical decision-making in complex patients rather than technical novelty alone. It suggests that, in selected patients with septic Charcot neuroarthropathy and severe systemic compromise, a staged strategy combining aggressive debridement, temporary circular external fixation, CLAP, and careful systemic monitoring may provide a potential limb-salvage option. Further studies are required to confirm the generalizability of this approach.

## 4. Limitations

This report has several limitations. First, it describes a single case, which limits generalizability. Second, although the clinical presentation strongly suggested neuropathic arthropathy with superimposed infection, histopathological confirmation was not performed, and the relative contribution of osteomyelitis and Charcot-related joint destruction could not be fully differentiated. Third, peripheral neuropathy was clinically inferred from the painless presentation but was not quantitatively assessed using formal neurophysiological testing. Fourth, functional outcomes were not fully assessed using standardized scoring systems. Although the AOFAS score at final follow-up was 90 points, indicating a favorable functional outcome, comprehensive patient-reported outcome measures such as the SF-36 were not evaluated. The presence of heart failure and associated fatigue in this patient may have limited the interpretability of general quality-of-life assessments. Overall, these limitations should be considered when interpreting the findings of this report.

## 5. Conclusions

Limb salvage may be feasible even in patients with septic Charcot midfoot involving the talonavicular and subtalar joints complicated by MRSA bacteremia and severe systemic compromise. In selected cases, a combination of aggressive debridement, continuous local antibiotic perfusion, and temporary external fixation may offer a potential strategy for infection control and preserve ambulation without major amputation.

## Figures and Tables

**Table 1:** Clinical timeline of treatment and outcomes.

Time Point	Clinical Course
Day 0	Admission; MRSA bacteremia confirmed; severe systemic condition
Day 1	Emergency debridement, circular external fixation, and initiation of CLAP
Postoperative Days 3–14	CLAP therapy; gradual improvement of local inflammatory findings
Postoperative Day 14	Resolution of local inflammation; normalization of WBC and CRP levels
Day 20	Second-stage surgery; external fixator removal; intraoperative cultures negative
Postoperative Weeks 3–5	Splint immobilization
After Week 5	Protected weight-bearing with patellar tendon-bearing (PTB) orthosis
2 months	Discharge without evidence of infection recurrence
4 years	Sustained infection-free survival; independent ambulation without orthosis

CLAP: continuous local antibiotic perfusion; WBC: white blood cell count; CRP: C-reactive protein.

## Data Availability

The original contributions presented in this study are included in the article. Further inquiries can be directed to the corresponding author.

## Abbreviations

BMI	Body mass index
CLAP	Continuous local antibiotic perfusion
eGFR	Estimated glomerular filtration rate
MRSA	Methicillin-resistant *Staphylococcus aureus*

## References

[1] Vopat M.L., Nentwig M.J., Chong A.C.M., Agan J.L., Shields N.N., Yang S.Y. (2018). Initial diagnosis and management for acute Charcot neuroarthropathy. Kans. J. Med..

[2] Kucera T., Shaikh H.H., Sponer P. (2016). Charcot neuropathic arthropathy of the foot: A literature review and single-center experience. J. Diabetes Res..

[3] Dardari D. (2020). An overview of Charcot’s neuroarthropathy. J. Clin. Transl. Endocrinol..

[4] Donegan R., Sumpio B., Blume P.A. (2013). Charcot foot and ankle with osteomyelitis. Diabet. Foot Ankle.

[5] Chantelau E. (2005). The perils of procrastination: Effects of early vs. delayed detection and treatment of incipient Charcot fracture. Diabet. Med..

[6] Wukich D.K., Sung W., Wipf S.A.M., Armstrong D.G. (2011). The consequences of complacency: Managing the effects of unrecognized Charcot feet. Diabet. Med..

[7] Hingsammer A.M., Bauer D., Renner N., Borbas P., Böni T., Berli M. (2016). Correlation of systemic inflammatory markers with radiographic stages of Charcot osteoarthropathy. Foot Ankle Int..

[8] Senneville É., Albalawi Z., van Asten S.A., Abbas Z.G., Allison G., Aragón-Sánchez J., Embil J.M., Lavery L.A., Alhasan M., Oz O. (2024). IWGDF/IDSA guidelines on the diagnosis and treatment of diabetes-related foot infections (IWGDF/IDSA 2023). Diabetes Metab. Res. Rev..

[9] Lipsky B.A., Berendt A.R., Cornia P.B., Pile J.C., Peters E.J.G., Armstrong D.G., Deery H.G., Embil J.M., Joseph W.S., Karchmer A.W. (2012). 2012 Infectious Diseases Society of America clinical practice guideline for the diagnosis and treatment of diabetic foot infections. Clin. Infect. Dis..

[10] Dalla Paola L., Brocco E., Ceccacci T., Ninkovic S., Sorgentone S., Marinescu M.G. (2009). Limb salvage in Charcot foot and ankle osteomyelitis: Combined use of single stage/double stage of arthrodesis and external fixation. Foot Ankle Int..

[11] Berli M., Vlachopoulos L., Leupi S., Böni T., Baltin C. (2017). Treatment of Charcot neuroarthropathy and osteomyelitis of the same foot: A retrospective cohort study. BMC Musculoskelet. Disord..

[12] Ramanujam C.L., Stuto A.C., Zgonis T. (2020). Surgical treatment of midfoot Charcot neuroarthropathy with osteomyelitis in patients with diabetes: A systematic review. J. Wound Care.

[13] Pinzur M.S. (2007). Neutral ring fixation for high-risk nonplantigrade Charcot midfoot deformity. Foot Ankle Int..

[14] Martin B., Chow J. (2021). The use of circular frame external fixation in the treatment of ankle/hindfoot Charcot neuroarthropathy. J. Clin. Orthop. Trauma.

[15] Ha J., Hester T., Foley R., Reichert I.L.H., Vas P.R.J., Ahluwalia R., Kavarthapu V. (2020). Charcot foot reconstruction outcomes: A systematic review. J. Clin. Orthop. Trauma.

[16] Alkhalfan Y., Lewis T.L., Kavarthapu V., Hester T. (2024). Investigation and management of diabetic foot osteomyelitis: An update for the foot and ankle orthopaedic surgeon. J. Clin. Orthop. Trauma.

[17] Game F.L., Selby N.M., McIntyre C.W. (2013). Chronic kidney disease and the foot in diabetes—Is inflammation the missing link?. Nephron Clin. Pract..

[18] Margolis D.J., Hofstad O., Feldman H.I. (2008). Association between renal failure and foot ulcer or lower-extremity amputation in patients with diabetes. Diabetes Care.

[19] Beaumont M., Hobson K., Rax H., Sutton A., Hill A., Lindström D., Dean A. (2025). Mortality and major amputation in patients with diabetes-related foot ulcers and chronic renal disease. ANZ J. Surg..

[20] Himeno D., Matsuura Y., Maruo A., Ohtori S. (2022). A novel treatment strategy using continuous local antibiotic perfusion: A case series study of a refractory infection caused by hypervirulent Klebsiella pneumoniae. J. Orthop. Sci..

[21] Nakajima H., Yamaguchi S., Kimura S., Horii M., Mikami Y., Sasho T., Ohtori S. (2025). Continuous local antibiotic perfusion for deep infection of the foot and ankle. J. Orthop. Sci..

[22] Armstrong D.G., Boulton A.J.M., Bus S.A. (2017). Diabetic foot ulcers and their recurrence. N. Engl. J. Med..

[23] Prompers L., Huijberts M., Schaper N., Apelqvist J., Bakker K., Edmonds M., Holstein P., Jude E., Jirkovska A., Mauricio D. (2008). Resource utilisation and costs associated with the treatment of diabetic foot ulcers. Prospective data from the Eurodiale Study. Diabetologia.

[24] Ceri H., Olson M.E., Stremick C., Read R.R., Morck D., Buret A. (1999). The Calgary Biofilm Device: New technology for rapid determination of antibiotic susceptibilities of bacterial biofilms. J. Clin. Microbiol..

[25] Choe H., Maruo A., Himeno D., Hieda Y., Inaba Y. (2026). Continuous local antibiotic perfusion: A novel technique for the treatment of orthopaedic infections: Narrative review. JBJS Open Access.

[26] Shimoda M., Choe H., Hieda Y., Abe K., Ike H., Mitsui H., Kono H., Kumagai K., Kobayashi N., Inaba Y. (2026). Factors associated with elevated serum gentamicin levels in hip and knee joints treated with continuous local antibiotic perfusion: A retrospective cohort study. J. Orthop. Sci..

